# Draft Genome Sequence of *Corynebacterium pseudodiphtheriticum* Strain 090104 “Sokolov”

**DOI:** 10.1128/genomeA.00921-13

**Published:** 2013-11-07

**Authors:** Andrey V. Karlyshev, Vyacheslav G. Melnikov

**Affiliations:** School of Life Sciences, Faculty of Science, Engineering and Computing, Kingston University, Kingston upon Thames, United Kingdoma; International Science and Technology Centre, Moscow, Russiab

## Abstract

This report describes the first draft genome sequence of a *Corynebacterium pseudodiphtheriticum* strain. The information on the genome organization and putative gene products will assist in better understanding of the molecular mechanisms involved in the beneficial probiotic effects of this bacterium.

## GENOME ANNOUNCEMENT

Here, we present a draft genome sequence of *Corynebacterium pseudodiphtheriticum* strain 090104 “Sokolov.” At the time of the preparation of materials for this publication, no complete or draft genome sequences of *C. pseudodiphtheriticum* were available.

*C. pseudodiphtheriticum* is a normal inhabitant of the nares and throat and could be a candidate for use as a nasal/throat probiotic. Bacteriological examinations of the nose and throat in diphtheria carriers showed cases of spontaneous replacement of *C. diphtheriae* by *C. pseudodiphtheriticum* (V. Melnikov, unpublished observation). Uehara et al. reported the elimination of nasal *Staphylococcus aureus* by application of *Corynebacterium* sp. (1). The negative association between *S. aureus* and corynebacteria suggests microbial competition during colonization (2, 3). These results were supported by recent research involving volunteers, in whom implantation of *C. pseudodiphtheriticum* strain 090104 resulted in the elimination of *S. aureus* (4).

Sequencing using a 314 chip and IonTorrent PGM produced 493,139 reads with a total size of 79.8 million bases. The IonTorrent Assembler plugin allowed assembly of 404,427 reads into 48 contigs ranging between 548 and 204,728 bases (*N*_50_, 86,796 bp) with a total size of 2,324,436 bases, corresponding to 28.78× genome coverage. The estimated genome GC content is 55.3%.

Annotation of the sequencing data was performed using the RAST server (5). This allowed the identification of 2,308 features, including 2,252 coding sequences, with the largest encoded protein of 3,102 amino acids being a putative acyl transferase involved in fatty acid biosynthesis.

Among the interesting features detected were genes encoding CRISPR-associated proteins such as Cas1, Cas2, and Csn1. These genes are known to be involved in bacterial resistance to invading bacteriophages (6). There are a number of genes contributing to resistance to antibiotics and other toxic compounds, such as vancomycin, fluoroquinolones, and beta-lactam antibiotics, as well as arsenate and heavy metals (cobalt-zinc-cadmium and mercury). The absence of genes required for polysaccharide transport suggests the lack of the ability to produce a polysaccharide capsule. Instead, the bacterium seems to have the potential to produce teichoic acid, which is a negatively charged cell wall polysaccharide with a backbone consisting of glycerol phosphate or ribitol phosphate residues linked via phosphodiester bonds. The presence of a protein similar to *Bacillus cereus* hemolysin may contribute to the pathogenicity of *C. pseudodiphtheriticum* in immunocompromised individuals (7).

The gene clusters encoding fimbrial subunits and sortase A (a protein involved in the attachment of fimbria to the cell surface) are flanked by transposase-encoding genes and may represent mobile elements required for bacterial attachment. The presence of two genes encoding homologues of the cell division inhibitor SulA suggests bacterial ability to enter a dormant (persister) state (8). No protein secretion systems or bacteriocin-related genes were found. Only a few phage-related genes were detected, suggesting the presence of a single prophage.

Identification of the genetic features of *C. pseudodiphtheriticum* strain 090104 will assist in better understanding of the mechanism of the probiotic action of this bacterium and in the development of new strains with improved properties.

### Nucleotide sequence accession numbers.

This whole-genome shotgun project has been deposited at DDBJ/EMBL/GenBank under the accession number AVFF00000000. The version described in this paper is version AVFF01000000.
